# Nitro-Glycerine

**Published:** 1880-05

**Authors:** 


### NITRO-GLYCERINE.

All of our readers are familiar with the *powerful* properties of this compound, but few are aware that its potency as a curative agent under the less alarming title of glonoin, is coming rapidly into favor as a powerful but temporary stimulant, both to the circulatory and nervous system. Its properties have been fully investigated by DeVritj, Muller, Mills, Defore, and others. It is freely soluble in alcohol and ether, and has a sweet, pungent, but not unpleasant taste. It is best dispensed in a one per cent. solution, of which from 5 to 20 m. may be administered at a dose.

A writer in the *British Medical Journal* says: It has been found that the effects of nitrite of amyl and nitro-glycerine on the pulse are similar. Both produce a marked dicrotism, and both accelerate the rapidity of the heart's action. They differ, however, in the time they respectively take to produce these effects. The full action of glonoin on the pulse is not observed until from two to six minutes after the dose has been taken; while in the case of nitrite of amyl the dicrotism appears in from 15 to 20 seconds after an inhalation, but its effect is transitory, being maintained for only a very short time. The glonoin acts more slowly, but the pulse does not resume the normal character for nearly half an hour.

